# Astrocyte dysfunction increases cortical dendritic excitability and promotes cranial pain in familial migraine

**DOI:** 10.1126/sciadv.aaz1584

**Published:** 2020-06-05

**Authors:** Jennifer Romanos, Dietmar Benke, Daniela Pietrobon, Hanns Ulrich Zeilhofer, Mirko Santello

**Affiliations:** 1 Institute of Pharmacology and Toxicology, University of Zurich, CH-8057 Zurich, Switzerland.; 2 Neuroscience Center Zurich, University of Zurich and ETH Zurich, CH-8057 Zurich, Switzerland.; 3 Department of Biomedical Sciences and Padova Neuroscience Center, University of Padova, 35131 Padova, Italy.; 4 CNR Institute of Neuroscience, Via Ugo Bassi 58/B, 35131 Padova, Italy.; 5 Institute of Pharmaceutical Sciences, ETH Zurich, CH-8093 Zurich, Switzerland.

## Abstract

Astrocytes are essential contributors to neuronal function. As a consequence, disturbed astrocyte-neuron interactions are involved in the pathophysiology of several neurological disorders, with a strong impact on brain circuits and behavior. Here, we describe altered cortical physiology in a genetic mouse model of familial hemiplegic migraine type 2 (FHM2), with reduced expression of astrocytic Na^+^,K^+^-ATPases. We used whole-cell electrophysiology, two-photon microscopy, and astrocyte gene rescue to demonstrate that an impairment in astrocytic glutamate uptake promotes NMDA spike generation in dendrites of cingulate cortex pyramidal neurons and enhances output firing of these neurons. Astrocyte compensation of the defective ATPase in the cingulate cortex rescued glutamate uptake, prevented abnormal NMDA spikes, and reduced sensitivity to cranial pain triggers. Together, our results demonstrate that impaired astrocyte function alters neuronal activity in the cingulate cortex and facilitates migraine-like cranial pain states in a mouse model of migraine.

## Teaser

In familial migraine, dysfunction of nonneuronal cells alters frontal cortex physiology and promotes cranial pain.

## INTRODUCTION

Astrocytes closely interact with neurons and strongly affect their functions at the synaptic, cellular, and circuit levels (*1*). Defective neuron-astrocyte interactions have been implicated in the establishment and development of several neurological disorders (*1*–*3*). Migraine is an extremely debilitating disease characterized by recurrent unilateral and severe headaches, frequently accompanied with several other neurological symptoms (*4*). However, migraine is much more than an episodic pain disorder. Several findings indeed suggest that migraine is a disease affecting a large part of the central nervous system and characterized by a global dysfunction in sensory information processing and integration, which also occurs between migraine episodes (interictal period) (*5*). For example, patients with migraine exhibit increased cortical responses to noxious and non-noxious sensory stimuli during the interictal period (*6*, *7*). At present, the cellular mechanisms responsible for these alterations are largely unknown. Astrocytes have been proposed to play a role in some inherited forms of migraine, including familial hemiplegic migraine type 2 (FHM2), an autosomal dominant form of migraine with aura. FHM2 is caused by mutations in the *Atp1a2* gene, which encodes the α_2_ subunit of the Na^+^, K^+^-dependent adenosine triphosphatase (ATPase) (α_2_ NKA) (*8*), an isoform that is almost exclusively expressed in astrocytes in the adult brain (*9*). Expression of the α_2_ NKA is reduced in the heterozygous *Atp1a2^+/R887^* FHM2 knock-in (KI) mice, which carry an *Atp1a2* missense mutation, causing a complete loss of function of recombinant α_2_ NKA (*8*, *10*). We previously showed that these FHM2 mice display impaired glutamate and K^+^ clearance by astrocytes of the primary somatosensory cortex, which, in turn, promotes cortical spreading depression (CSD), the neuronal correlate of the aura symptoms that precede migraine headache (*3*).

In this study, we took advantage of the FHM2 KI mouse model to understand whether and how a mutation in an astrocyte-specific protein affects neuronal functions in the cingulate cortex (Cg). This cortical region is crucial for pain processing and displays altered functionality in patients with migraine (*7*, *11*–*13*). We show that impairment in astrocytic glutamate uptake in this region strongly enhances cortical dendritic excitability, especially the generation of *N*-methyl-d-aspartate (NMDA) spikes in layer 5 (L5) pyramidal neurons, and enhances their output firing. Moreover, we reveal that FHM2 mice display increased sensitivity to head pain triggers. Last, we show that rescuing the disease-causing mutation in astrocytes of the Cg recovers neuronal function and reduces the pain phenotype. Our results provide a clear example of how astrocyte dysfunction produced by a genetic defect affects neuronal activity in the Cg and affects sensitivity to head pain triggers.

## RESULTS

### Astrocytic dysfunctions in the Cg of FHM2 mice

**Fig. 1:**
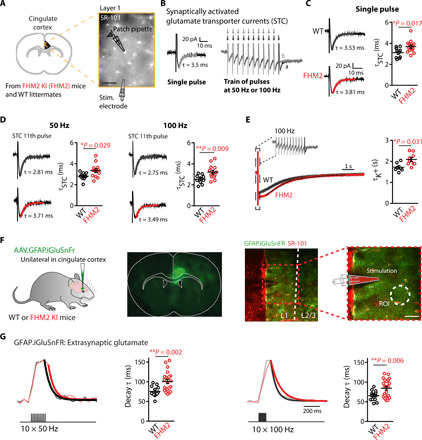
Aberrant astrocytic glutamate and K^+^ uptake in the Cg of FHM2 mice. (**A**) Schematic representation of the experiment. Scale bar, 30 μm. (**B**) Superimposed representative traces of the inward current evoked in an astrocyte with different stimulation patterns. (**C**) The decay time of inward currents evoked by single-pulse stimulation is slower in FHM2 mice (red) compared to WT mice (black). (**D**) The average STC decay times of the last pulse of the trains at 50 (left) and 100 Hz (right) are significantly slower in FHM2 mice. Each point represents the STC decay time in one astrocyte. (**E**) Decay kinetics of the K^+^ inward current following trains of 100 Hz stimulation are slower in FHM2 mice. (**F**) Injection of AAV.GFAP.iGluSnFr unilaterally in the Cg of WT and FHM2 mice. A typical two-photon experiment showing the expression of iGluSnFr on astrocytes in the Cg (green) and sulforhodamine 101 dye (SR-101; red) is shown. The theta glass electrode for synaptic stimulation is placed in the inner L1, and glutamate is imaged from an ROI adjacent to the electrode. Scale bar, 40 μm. (**G**) Upon trains of synaptic stimulation (10 × 50 Hz and 10 × 100 Hz), robust and reliable increases in iGluSnFr emission could be detected. The decay kinetics of the averaged transients are slower in FHM2 mice. Representative traces are the average of at least five sweeps. Data are means ± SEM. Two-tailed unpaired *t* test was used.

The α_2_ NKA is physically and functionally coupled to glutamate transporters (GluTs) expressed on perisynaptic astrocyte processes (*9*, *14*). The reduced expression of α_2_ NKA in heterozygous *Atp1a2^+/R887^* mice (FHM2 mice) results in a reduction in glutamate and K^+^ buffering capacity of astrocytes in the primary somatosensory cortex (*3*, *10*). To investigate FHM2-associated alterations in glutamate and K^+^ uptake in the Cg, a brain region crucially involved in pain processing, we performed whole-cell patch-clamp recordings from astrocytes in acute cortical slices (Fig. 1A). We recorded synaptically evoked GluT currents (STCs) and K^+^ uptake currents induced by electrical stimuli to L1 afferent fibers with an extracellular electrode and applied different stimulation paradigms (single pulses and pulse trains at 50 and 100 Hz; Fig. 1, A and B). Glutamate uptake was significantly slower in the Cg of FHM2 mice compared to their wild-type (WT) littermates, which was reflected by higher STC decay time constants (τ) [Fig. 1, C and D; single pulse: WT τ_decay_ = 3.11 ± 0.15 ms (*n* = 10 cells, *N* = 5 mice) and FHM2 τ_decay_ = 3.69 ± 0.15 ms (*n* = 14, *N* = 8; *P = 0.017); 50 Hz: WT τ_decay_ = 2.81 ± 0.11 ms (*n* = 10) and FHM2 τ_decay_ = 3.34 ± 0.18 ms (*n* = 13; *P = 0.029); 100 Hz: WT τ_decay_ = 2.54 ± 0.13 ms (*n* = 10) and FHM2 τ_decay_ = 3.22 ± 0.18 ms (*n* = 13; **P = 0.009)]. The decay kinetics of the K^+^ currents following a train of pulses at 100 Hz were also significantly slower in the FHM2 mice [Fig. 1E; WT τ_decay_ = 1.72 ± 0.08 s (*n* = 7 cells, *N* = 4 mice) and FHM2 τ_decay_ = 2.25 ± 0.18 s (*n* = 10, *N* = 5 mice; *P = 0.03)]. We observed no difference in astrocytic resting membrane potential nor input resistance between the two groups (fig. S1). These data indicate that in the Cg of adult FHM2 mice, astrocytic uptake of neuron-derived glutamate and K^+^ was impaired.

Slower glutamate clearance by astrocytes may lead to prolonged and increased glutamate levels in the extracellular space. To directly test this prediction, we took advantage of the intensity-based glutamate fluorescent sensor iGluSnFr (*15*) that we expressed on the extracellular side of the astrocytic plasma membrane (Fig. 1F and see Materials and Methods). Two-photon imaging of iGluSnFr glutamate signals allowed the study of the time course of extracellular glutamate following synaptic stimulation. Synaptic activity was evoked by focal electrical stimulation in L1 of the Cg, and glutamate was imaged in a region of interest (ROI) in the proximity of the stimulation electrode (Fig. 1F). To estimate the speed of glutamate clearance, we fitted the decay of the averaged evoked glutamate transients (10 trials) with a monoexponential curve (*16*). We found that synaptically evoked iGluSnFr transients in the adult Cg were sensitive to minor changes in the activity of astrocytic GluTs. Accordingly, partial blockade of GluTs with subsaturating concentrations of threo-beta-Benzyloxyaspartate (DL-TBOA) (3 μM), a concentration that mimics in WT mice the slowing of STC decay kinetics produced by the FHM2 mutation (*3*), increased the decay time constant of extracellular glutamate transients in WT mice (fig. S2).

We subsequently recorded iGluSnFr signals in the Cg of FHM2 mice and compared it with WT littermates. The iGluSnFr signals displayed a longer time course following trains of 50 and 100 Hz stimulations in the FHM2 mice, which were 32 and 29% slower, respectively [Fig. 1G; 50 Hz: WT τ_decay_ = 75.56 ± 3.62 ms (*n* = 13 slices, *N* = 5 mice) and FHM2 τ_decay_ = 100.9 ± 5.33 ms (*n* = 22, *N* = 7; **P = 0.0019); 100 Hz: WT τ_decay =_ 65.6 ± 3.32 ms (*n* = 14) and FHM2 τ_decay_ = 84.57 ± 4.77 ms (*n* = 22; **P = 0.006)]. Note that we and others have previously demonstrated that the size and location of ROIs, stimulation intensity, and sulforhodamine 101 dye (SR-101) do not influence the iGluSnFr decay kinetics (*16*, *17*). Overall, these experiments demonstrate that in the adult Cg, astrocytes carrying the W887R α_2_ NKA mutation responsible for FHM2 display defective glutamate and K^+^ buffering capacity upon repetitive synaptic stimulation, which results in temporally prolonged glutamate spillover.

### Facilitation of NMDA spike generation in L5 pyramidal neurons of FHM2 mice

**Fig. 2:**
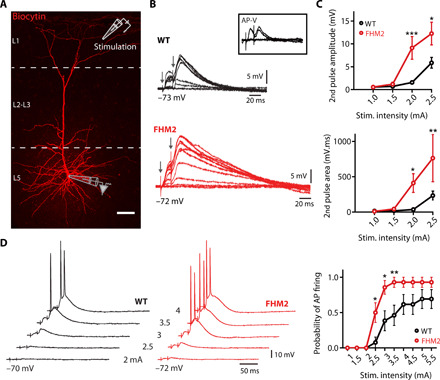
Facilitation of NMDA spike generation in L5 pyramidal neurons in the Cg of FHM2 mice. (**A**) Image of whole-cell recording from the soma of a biocytin-labeled L5 pyramidal cell in the Cg showing the location of the recording pipette (L5) and stimulation electrode (in close proximity to single branches of tuft dendrites in L1 of the Cg). Scale bar, 50 μm. (**B**) Representative traces of NMDA spikes evoked by focal synaptic stimulation (paired pulse, 50 Hz) of increasing stimulation intensities that cause an abrupt and nonlinear increase in amplitude and AUC of the second pulse, which is characteristic of NMDA spikes. This nonlinear increase is abolished in the presence of NMDA receptor blocker AP-V (50 μM; box). (**C**) The amplitude and the AUC of the second pulse are significantly higher in FHM2 mice compared to WT mice upon lower stimulation intensities. (**D**) Paired-pulse stimulation had a higher probability to evoke a somatic action potential (AP) in FHM2 mice. Data are means ± SEM. Two-way analysis of variance (ANOVA) with Bonferroni post hoc test and *Z* score were used.

Pharmacological reduction of astrocytic glutamate uptake capacity in the cortex increases extracellular buildup of glutamate, which directly affects NMDA receptor activation in L5 pyramidal neurons (*17*). In addition, glutamate spillover promotes the occurrence of NMDA dendritic spikes (*18*). These spikes are local events (depolarizations) caused by the regenerative and voltage-dependent activation of NMDA receptors in specific dendritic branches and have been shown to strongly promote pyramidal cell firing in vivo (*19*). We tested whether the alteration of astrocytic glutamate clearance in FHM2 mice would affect NMDA spike generation. We evoked NMDA spikes in the distal dendrites of L5 pyramidal neurons by focal synaptic stimulation (paired pulse, 50 Hz) in close proximity to single branches of tuft dendrites in L1 of the Cg (Fig. 2A). Increasing stimulation intensities caused a nonlinear increase in the amplitude and area under the curve (AUC) of the second pulse, which is characteristic of NMDA spikes (*20*). This nonlinear increase was abolished in the presence of NMDA receptor blocker d,l-2-amino-5-phosphonovaleric acid (AP-V) (Fig. 2B).

As predicted from the reduced glutamate clearance in FHM2 mice, these mice displayed facilitated NMDA spike generation reflected by higher amplitude and AUC of the voltage responses evoked by L1 synaptic stimulations [Fig. 2C; second pulse amplitude: WT, 1.56 ± 0.4 mV (*n* = 12 cells, *N* = 8 mice) and FHM2, 9.13 ± 2.48 mV (*n* = 13, *N* = 6; ***P < 0.0001); second pulse AUC: WT, 40.64 ± 13.9 mV ms (*n* = 11) and FHM2, 411.5 ± 131 mV ms (*n* = 12; *P < 0.05); stimulation intensity, 2 mA]. Partial blockade of GluTs with subsaturating concentrations of the GluT blocker dl-TBOA (3 μM) significantly enhanced NMDA spike generation in L5 pyramidal neurons in WT mice (fig. S3), mimicking the effect of the FHM2 genetic mutation.

Dendritic NMDA spikes have been reported to promote somatic firing in neurons of the somatosensory cortex (*19*). The main apical dendrite of L5 pyramidal neurons of the Cg displays a remarkably low dendrite-to-soma attenuation of slow synaptic inputs compared to other cortical regions (*21*), suggesting that NMDA-mediated dendritic depolarizations may have an even stronger influence on somatic depolarization and action potential (AP) firing in this region. We find that our NMDA spike induction protocol easily triggered somatic firing (Fig. 2D). Consequently, we predicted that the facilitation of NMDA spikes that we identified in FHM2 Cg L5 pyramidal neurons should lead to increased firing of these neurons. We found that the probability of AP firing following NMDA spikes was significantly higher in FHM2 mice compared to their WT littermates (Fig. 2D). These data demonstrate that in the Cg, defective glutamate uptake and the prolonged presence of synaptically released glutamate are accompanied by a facilitation in NMDA spikes and somatic firing of L5 pyramidal cells.

**Fig. 3:**
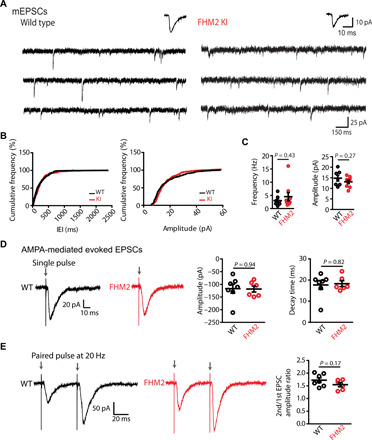
Baseline synaptic activity is similar in L5 pyramidal cells of FHM2 and WT mice. (**A**) Representative example traces (6 s) of whole-cell mEPSCs recordings from WT mice (left) and FHM2 KI mice (right). Average mEPSCs are shown above the traces. (**B**) Cumulative frequency plots of interevent intervals (IEI) (left) from WT and FHM2 KI and cumulative frequency plot of mEPSCs amplitudes from the same cells (right) calculated from the presented traces above. (**C**) No difference is observed in mEPSCs frequency between WT and FHM2 KI slices. Data points represent individual cells. (**D**) Left: Example traces of AMPA-mediated evoked EPSCs following a single pulse in WT (black) and FHM2 mice (red). Right: The amplitude of EPSCs following a single pulse is similar in WT and FHM2 mice. Decay kinetics are also similar in WT and FHM2 KI mice. (**E**) Left: Example traces of AMPA-mediated evoked EPSCs following a paired-pulse stimulation at 20 Hz in WT and FHM2 mice. Right: The second-to-first EPSC amplitude ratio is not different in WT compared to FHM2 mice. Data are means ± SEM. Two-tailed unpaired *t* test was used.

We then investigated whether synaptic activity is altered in FHM2 mice. We first recorded miniature excitatory postsynaptic currents (mEPSCs) from L5 pyramidal cells of WT and FHM2 mice (Fig. 3, A, B, and C) and found no change in baseline synaptic activity (mEPSC frequency and amplitude) between the two groups [mEPSC frequency: WT, 3.02 ± 0.71 Hz (*n* = 7 cells) and FHM2 KI, 4.53 ± 1.54 Hz (*n* = 9 cells; *P* = 0.43); mEPSC amplitude: WT, 14.81 ± 1.35 pA and FHM2 KI, 13.09 ± 0.82 pA (*P* = 0.27)]. We additionally recorded AMPA-mediated EPSCs evoked with single-pulse and paired-pulse extracellular synaptic stimulations (Fig. 3, D and E). Upon single-pulse stimulations, both the amplitude and decay kinetics of EPSCs were similar in WT and FHM2 mice [amplitude: WT, −117 ± 18.04 pA (*n* = 7 cells) and FHM2 KI, −118.6 ± 12.03 pA (*n* = 6 cells; *P* = 0.94); decay: WT, 17.7 ± 2.1 ms (*n* = 7 cells) and FHM2 KI, 18.29 ± 1.4 ms (*n* = 6 cells; *P* = 0.82)]. Similarly, upon paired-pulse stimulation at 20 Hz, the second-over-first EPSC amplitude ratio was comparable in WT and FHM2 mice [second/first amplitude ratio: WT, 1.73 ± 0.09 (*n* = 7 cells) and FHM2, 1.54 ± 0.08 (*n* = 7 cells; *P* = 0.17)]. These datasets strongly argue against alterations in glutamate release or in AMPA-mediated synaptic transmission in the Cg of FHM2 mice.

### Local astrocytic defects are responsible for NMDA-mediated neuronal dysfunctions

**Fig. 4:**
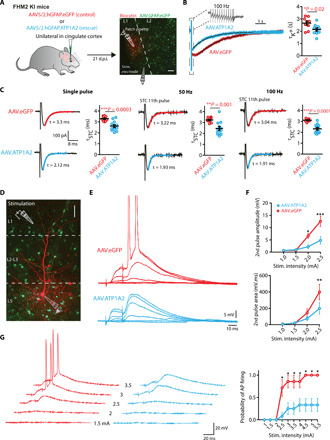
Local astrocytic defects are responsible for NMDA-mediated neuronal dysfunctions. (**A**) AAVs containing the WT form of *Atp1a2* (AAV5/2.hGFAP.ATP1A2) or a control virus (AAV5/2.hGFAP.eGFP) is unilaterally injected in the Cg of FHM2 mice. At 21 d.p.i., astrocytes expressing either GFP or Atp1a2 in L1 of the Cg cortex are targeted for recording in acute brain slices while stimulating nearby neurons. Scale bar, 40 μm. (**B**) Example traces of the K^+^ current following trains of 100 Hz stimulations in FHM2 mice injected with the control virus (red) and those injected with the rescue virus (blue). The decay kinetics of K^+^ currents are significantly faster in rescued FHM2 mice compared to control FHM2 mice. (**C**) Upon all stimulation patterns (single pulse and trains of 50 and 100 Hz), the decay kinetics of STCs are faster in rescued mice compared to the control group. (**D**) Image of whole-cell recording from the soma of a biocytin-labeled L5 pyramidal cell in the Cg surrounded by eGFP-expressing astrocytes. Scale bar, 40 μm. (**E**) Representative traces of NMDA spikes evoked by focal synaptic stimulation (paired pulse, 50 Hz) in control FHM2 mice (red) and in rescued FHM2 mice (blue). (**F**) Both the amplitude and the AUC of the second pulse are significantly lower in rescued FHM2 mice compared to the control group. (**G**) Paired-pulse stimulation had a lower probability to evoke a somatic AP in rescued FHM2 mice (blue) compared to control mice (red). Data are means ± SEM. Two-tailed unpaired *t* test, two-way ANOVA with Bonferroni post hoc test, and *Z* score were used.

Excitatory neuronal activity appears to be highly increased in the Cg of FHM2 mice. Nevertheless, to what extent the local astrocytic malfunction is responsible for the modifications in neuronal activity is not clear. To address this question, we compensated for the astrocyte dysfunction in the Cg region of FHM2 mice by expressing the WT form of α_2_ NKA (*Atp1a2*) in astrocytes. To deliver *Atp1a2*, we used an adeno-associated virus (AAV) of the 5/2 serotype that preferentially targets astrocytes and took advantage of the astrocyte promoter hGFAP (AAV.hGFAP.ATP1A2) (*22*). As a control, AAV of the same serotype and with the same vector backbone, but containing only enhanced green fluorescent protein (eGFP) (AAV.hGFAP.eGFP), was injected (Fig. 4A; also see Materials and Methods). Since AAV vectors’ size is limited to ~4.7 kb, it was not possible to include a reporter gene in the virus with ATP1A2. Therefore, to visualize the injection site, we injected a mixture of 0.5 μl of the two viruses with a ratio of 2:1 of AAV.hGFAP.ATP1A2 to the control virus (AAV.hGFAP.eGFP). Two to 3 weeks following injections, immunohistochemistry experiments showed that AAV.hGFAP targeted astrocytes with no apparent neuronal expression (fig. S4). Consistent with previous findings on other brain regions (*10*), the Cg of FHM2 mice showed a substantial reduction of α2 NKA expression levels (about 70%) compared to WT littermates (fig. S5A). This reduction was significantly, albeit not fully, restored in FHM2 mice injected with the rescue virus (fig. S5A). The Western blot experiments were performed 15 days post-injection (d.p.i.) of the viruses. Accordingly, the functional recovery of the STC decay kinetics was also incomplete at 15 d.p.i. (fig. S6). This may, at least in part, account for the incomplete restoration of α2 NKA expression. For this reason, the functional and behavioral experiments (see later) were performed mostly at 21 d.p.i., when the STC decay kinetics became similar to WT levels (fig. S6). In addition, it is also likely that the Cg tissue extracted for Western blot analysis contained regions with a mixture of high and low (or no) virus expression.

We additionally evaluated the expression levels of the astrocytic GluTs, GLT-1 and GLAST, that both play a crucial role in glutamate uptake in the Cg (*17*). We observed a 25% reduction of GLAST expression in the Cg of FHM2 mice, which was restored to WT levels in FHM2 mice injected with the rescue virus (fig. S5B). On the other hand, no changes in GLT-1 expression levels were observed between the different groups (fig. S5C).

We then performed electrophysiological recordings in the same manner as in Fig. 1 from FHM2 astrocytes either expressing WT *Atp1a2* or the eGFP control. Our data show that both K^+^ [Fig. 4B; control τ_decay_ = 2.66 ± 0.16 s (*n* = 9 cells, *N* = 4 mice) and rescue τ_decay_ = 2.14 ± 0.13 s (*n* = 10, *N* = 4; *P = 0.02)] and active glutamate clearance by astrocytes became significantly faster in FHM2 mice in which WT *Atp1a2* was expressed compared to FHM2 mice that were injected with the control virus [Fig. 4C; single pulse: control τ_decay_ = 3.3 ± 0.06 ms (*n* = 10 cells, *N* = 4 mice) and rescue τ_decay_ = 2.6 ± 0.13 ms (*n* = 12, *N* = 4; ***P = 0.0003); 50 Hz: control τ_decay =_ 3.24 ± 0.06 ms (*n* = 10) and rescue τ_decay_ = 2.46 ± 0.18 ms (*n* = 12, **P = 0.001); 100 Hz: control τ_decay =_ 3.11 ± 0.04 ms (*n* = 10) and rescue τ_decay_ = 2.31 ± 0.12 ms (*n* = 12; ***P = 0.0001)]. The decay kinetics of the uptake currents in the rescued FHM2 mice became comparable to those observed in WT mice (fig. S7A). The astrocytic resting membrane potential was slightly but significantly hyperpolarized in rescued FHM2 mice compared to those injected with control virus, with no difference in input resistance (fig. S8).

In light of these results, we investigated whether the rescue of the astrocytic dysfunctions could indeed affect the neuronal defects observed in FHM2 mice. To this end, we evoked NMDA spikes in the distal dendrites of L5 pyramidal neurons by focal synaptic stimulation as in previous experiments (Fig. 4, D and E). The amplitude and AUC of NMDA spikes were significantly lower in FHM2 mice injected with WT *Atp1a2* compared to FHM2 mice injected with the control virus [Fig. 4F; second pulse amplitude: control, 12.56 ± 2.11 mV (*n* = 7 cells, *N* = 3 mice) and rescue, 4.8 ± 1.52 mV (*n* = 11, *N* = 4; ***P < 0.0001); second pulse AUC: control, 432.7 ± 87.7 mV ms (*n* = 7) and FHM2, 198.15 ± 69 mV ms (*n* = 12; **P < 0.01); stimulation intensity, 2.5 mA]. The values in the rescued FHM2 mice were comparable to those in WT mice (fig. S7B). This rescue was also accompanied by a significantly lower probability of AP firing following NMDA spikes in the rescued FHM2 mice (Fig. 4G).

### Local astrocyte dysfunction in the Cg influences orofacial pain in FHM2 mice

**Fig. 5:**
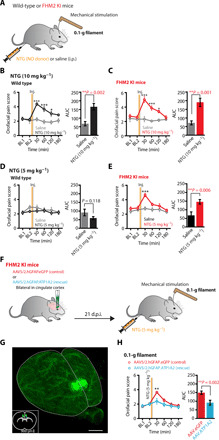
Local rescue of astrocyte dysfunction in the Cg reduces facial pain in FHM2 mice. (**A**) Schematic illustration of the experimental design. (**B**) Time course showing the orofacial pain score in WT mice injected with either saline (gray) or NTG (black). NTG (10 mg kg^−1^) elicits hypersensitivity to touch at 30, 60, and 120 min following injection. (**C**) Same as (B) but for FHM2 mice injected with saline (gray) or NTG (red). NTG (10 mg kg^−1^) evokes higher orofacial pain scores at 30, 60, and 120 min following injection. (**D**) A lower dose of NTG (5 mg kg^−1^) does not elicit a higher sensitivity in WT mice. (**E**) In FHM2 mice, NTG (5 mg kg^−1^) evokes a higher orofacial pain score at 30 min after injection. (**F**) Schematic illustration of the experimental design. (**G**) Three-dimensional reconstruction of a cleared brain imaged with light-sheet microscopy showing the volume and site of the injection in Cg after ex vivo fixation. Scale bar, 300 μm. The bottom left image shows a single coronal section, and dots indicate the center of the injection volume of the viruses from six brains. (**H**) Time course showing the orofacial pain score in FHM2 mice injected with the control virus (red) or the rescue virus (blue). NTG (5 mg kg^−1^) only evoked a higher orofacial pain score at 30 min after injection in control FHM2 mice and not in rescued FHM2. Data are means ± SEM. Two-way ANOVA with Bonferroni post hoc test and two-tailed unpaired *t* test were used.

The Cg is a critical cortical region in encoding cephalic pain. Altered neuronal activity in this brain area has been reported to influence the activation and sensitization of pain pathways in pathological pain conditions (*23*). Whether local astrocyte dysfunction in the Cg can facilitate cranial pain in FHM2 mice is unknown. To explore this possibility, we activated the cranial pain pathway via a single systemic injection of the nitric oxide donor nitroglycerin (NTG). NTG is considered a reliable cranial pain trigger especially in migraine-susceptible patients. Only in patients with migraine, NTG induces a delayed migraine-like headache with associated features (e.g., premonitory symptoms) that resemble their own spontaneous migraine attacks (*24*). Systemic injections of NO donors have also been used in rodents (*25*) and have been shown to evoke hypersensitivity to touch (typical migraine symptom), particularly in mice carrying a mutation associated with migraine with aura (*26*). To assess the development of facial mechanical sensitization upon NTG injections, we gently poked the mice in the orofacial region with von Frey filaments and scored the evoked nocifensive behavior (orofacial pain score; see Materials and Methods). This scoring system has been previously used to assess trigeminal neuropathic pain in rodent models (*27*). We first found that NTG [10 mg kg^−1^, intraperitoneally (i.p.)] triggered facial mechanical hypersensitivity in both WT and FHM2 mice at 30, 60, and 120 min post NTG injection compared to mice injected with saline [Fig. 5, B and C; AUC for WT: saline, 67.56 ± 12.50 (*N* = 6 mice) and NTG, 165.5 ± 19.08 (*N* = 6; **P = 0.0016); AUC for FHM2: saline, 71.32 ± 11.72 (*N* = 6) and NTG, 190.3 ± 23.92 (*N* = 6; **P = 0.0012)]. FHM2 mice developed orofacial hypersensitivity upon NTG doses that were ineffective in WT littermates [5 mg kg^−1^; Fig. 5, D and E; AUC for WT: saline, 91.51 ± 19.11 (*N* = 6 mice) and NTG, 58.90 ± 8.88 (*N* = 8; *P* = 0.12); AUC for FHM2: saline, 67.16 ± 20.29 (*N* = 7) and NTG, 146.0 ± 13.13 (*N* = 7; **P = 0.0068)]. These results suggest that the FHM2 mutation promotes the development of NTG-induced facial mechanical hypersensitivity. The same phenotype was observed with a lighter von Frey filament of 0.025 g (fig. S9). Activity tests showed no difference between saline- and NTG-treated mice or between FHM2 and WT littermates, suggesting that the different responses to mechanical stimulation were not accompanied by alterations in motor function (fig. S10, A and B).

Since the Cg is implicated in pain signaling and development of mechanical hypersensitivity in several pathological pain syndromes, we wondered whether rescuing local astrocyte dysfunction in this brain area could ameliorate the increased nocifensive responses detected in FHM2 mice upon NTG treatment (*23*). To that end, we expressed WT *Atp1a2* (AAV.hGFAP.ATP1A2) in Cg astrocytes of FHM2 mice. As a control, FHM2 mice were injected with AAV.hGFAP.eGFP. Three weeks after viral injections, we triggered facial mechanical sensitivity by NTG injections (5 mg kg^−1^) and performed facial von Frey tests as described above (Fig. 5, F and G). We found that rescuing the astrocytic loss-of-function FHM2 mutation locally in the Cg was sufficient to attenuate the acute hypersensitive phenotype induced by NTG in FHM2 mice (Fig. 5H and fig. S9), suggesting that the local astrocyte dysfunction in the Cg is implicated in orofacial pain sensitivity [AUC: control, 148.8 ± 9.62 (*N* = 14 mice) and rescue, 91.40 ± 13.98 (*N* = 13 mice; **P = 0.0021)].

Together, our results demonstrate that astrocytes in a genetic migraine model display altered glutamate and K^+^ clearance in the Cg, which facilitate neuronal NMDA spike generation and synaptically evoked AP firing. We report that these alterations are pathologically relevant since local rescue of the astrocytic dysfunctions reduces facial hypersensitivity induced by a migraine-relevant pain trigger.

## DISCUSSION

### Astrocytic dysfunction enhances NMDA-mediated dendritic excitability in Cg of FHM2 mice

In this study, we first confirmed that in the adult Cg of FHM2 mice, similar to the developing somatosensory cortex (*3*), α_2_ NKA dysfunction impairs both K^+^ and synaptically released glutamate uptake by astrocytes. Western blot analysis performed in FHM2 mice also revealed a significant reduction in the expression levels of α2 NKA and the GluT GLAST, which we previously reported to be implicated in astrocyte-mediated glutamate uptake in this cortical region (*17*). The reduced GLAST expression in FHM2 Cg could suggest potential physical coupling between α2 NKA and GLAST in Cg astrocytes, as was previously shown to occur between α2 NKA and GLT-1 in perisynaptic astrocytic processes of the somatosensory cortex (*9*). As a consequence of this tight coupling, a reduced density of GLT-1 transporters in perisynaptic astrocytic processes could be previously detected with electron microscopy in the developing barrel cortex of FHM2 mice (*3*). A local reduction of GLT-1 density specifically around synapses may remain undetected in Western blots and be consistent with the unaltered expression of GLT-1 in FHM2 Cg (*28*). With regard to the reported impairment of K^+^ clearance in the Cg of FHM2 mice, this is likely to be directly ascribed to the reduced α2 NKA expression and function, which plays a major role in K^+^ clearance following trains of high-frequency stimulation (*29*).

The slowdown in glutamate clearance by astrocytes prolonged the presence of elevated glutamate levels in the extracellular space. Increase in glutamate spillover enhances cortical excitatory neurotransmission, particularly NMDA receptor–mediated transmission (*17*). Consistently, we found a facilitation of NMDA spike generation in tuft dendrites of L5 pyramidal cells both in FHM2 mice and upon subsaturating concentrations of the GluT blocker dl-TBOA (that reduces glutamate clearance to an extent similar to that produced by the FHM2 mutation) in WT mice. The probability of AP firing following NMDA spikes was also increased in FHM2 mice. In contrast, no changes were observed in spontaneous and evoked synaptic transmission in FHM2 mice.

To establish a causal relationship between the astrocyte malfunction and the observed neuronal modifications in FHM2, we compensated for the astrocyte dysfunction by expressing the WT form of the *Atp1a2* gene in the Cg of FHM2 mice. This intervention reversed the defective glutamate and K^+^ clearance by astrocytes.

Compensating the astrocyte dysfunction reduced the facilitation of dendritic NMDA spike generation, thereby lowering the output firing induced by these spikes. These findings demonstrate that NMDA spike generation on tuft dendrites of L5 pyramidal cells and the subsequent neuronal output are directly and dynamically affected by astrocytic dysfunction.

NMDA spikes are believed to be the dominant mechanism by which distal synaptic inputs lead to firing of pyramidal neurons in the cortex (*30*). Since dendritic spikes increase the computational properties of individual neurons (*19*, *30*), the facilitation of NMDA spike generation and the resulting firing of L5 pyramidal cells (the main output cells of the Cg) could greatly influence network activity in downstream cortical and subcortical areas involved in pain processing.

### The altered cellular functions in the Cg of FHM2 mice lead to hypersensitivity to a migraine-relevant pain trigger

In migraineurs, NTG administration induces a delayed migraine-like headache with associated features such as premonitory symptoms and allodynia (*24*). NTG-induced hyperalgesia in animals provides a behavioral model of the NTG-induced allodynia observed in migraineurs during the attack (*25*). Using this model, we found that relatively low doses of NTG trigger hypersensitivity to facial mechanical stimulation in FHM2 mice, while they are ineffective in WT mice. A similar finding was previously reported for another genetic mouse model of migraine (*26*). Local rescue of the astrocytic defect by expressing the WT *Atp1a2* gene in the Cg of FHM2 mice strongly reduced their increased nociceptive response upon NTG treatment. This finding is consistent with the conclusion that the hypersensitivity of FHM2 mice to a migraine-relevant trigger is largely due to altered neural function in the Cg.

### The Cg involvement in migraine pathophysiology

The anterior Cg (ACC) and midcingulate cortex (MCC) play a key role in pain processing (*23*, *31*). The ACC is consistently activated in humans and animal models upon nociceptive stimuli (*32*), and neuronal plasticity in the ACC is correlated with the development of chronic pain (*21*, *23*). The ACC is also among the regions that are activated during spontaneous migraine attacks (*33*) and during the premonitory phase of the delayed migraine-like headache induced by NTG infusion in migraineurs (*34*). Moreover, functional imaging studies show increased activation of both the ACC (*12*) and the MCC (*13*, *35*) in response to noxious (including trigeminal) stimulation in migraineurs during the interictal period. Migraineurs show increased activation of the MCC that correlates with increased pain rating (sensitization) during repeated trigeminal noxious stimulation, while healthy controls show decreased MCC activation and pain rating (habituation) (*13*). However, the underlying mechanism of this hyperactivity and its potential involvement in cranial pain induction was still elusive. We report an astrocyte-mediated facilitation of NMDA spike generation and a subsequent increase of L5 pyramidal cell firing in the Cg of FHM2 mice, which may be potentially involved in pain generation and/or sensitization of the pain processing system in familial migraine. The evidence that astrocyte dysfunction in the Cg can specifically increase facial tactile sensitivity to a pain trigger supports the notion that the Cg is a critical hub in pain processing and may additionally gate the activation of cranial pain pathways. Whether the ACC, the MCC, or both mediate this remains unclear. The ACC is broadly connected to the salience network and projects to the periaqueductal gray, rostral ventromedial medulla, and dorsal horn (*5*). An interesting pathway from the MCC to the posterior insula (and involving descending serotoninergic facilitation of nociception from the raphe magnus nucleus) has been shown to be necessary and sufficient for the induction and maintenance of pain sensitization and could also be involved in the observed behavior (*31*). Therefore, heightened activity in the ACC and/or MCC could have broad effects on migraine-relevant pain perception and descending modulatory circuits, which could lead to a loss of pain inhibition and/or to pain facilitation. It would therefore be intriguing to test how the rescue of the astrocytic mutation in the ACC or MCC affects the activity of those downstream regions crucial for the development or sensitization of cranial pain.

Our data support the idea that FHM2-associated astrocytic dysfunction in particular brain regions may engender different migraine-relevant functional consequences. In the somatosensory cortex, it lowers the CSD threshold with a potential impact on aura occurrence and cranial nociceptor activation (*3*, *10*). CSD facilitation is a common feature of all genetic models of migraine that have been investigated so far (*26*, *36*, *37*). In the hippocampus, the FHM2 mutation causes abnormal region-dependent synaptic plasticity, which might underlie some of the memory deficits observed in FHM2 patients (*38*). We demonstrate instead that Cg astrocyte dysfunction in FHM2 mice leads to hypersensitivity to a migraine-relevant trigger.

In conclusion, we provide evidence that astrocyte dysfunction in the Cg, a nonsensory cortical area, is implicated in heightened sensitivity to head pain triggers and may be involved in pain generation and/or sensitization of the pain processing system in familial migraine. Understanding the cellular and molecular nature of circuit-specific network dysfunctions associated with familial migraine might be key to shed light on incompletely understood aspects of migraine pathophysiology.

## MATERIALS AND METHODS

### Animals

Experiments were performed using adult (5 to 9 weeks old) heterozygous KI mice harboring the W887R FHM2 mutation [*Atp1a2^+/R887^* mice; (*10*)] and their WT littermates (background C57BL/6J; male and female in equal or near-equal number). Adult mice were group-housed up to five in filter-top cages with a standard 12-hour light/12-hour dark cycle and food and water available ad libitum. Permission for animal experiments was obtained from the Tierversuchskommission of the canton of Zurich, Zurich, Switzerland. All animal experiments complied with the relevant ethical regulations.

### Chemicals and drugs

Reagents for artificial cerebrospinal fluid (ACSF) and internal solutions, biocytin, 6-nitro-7-sulfamoylbenzo[*f*]quinoxaline-2,3-dione (NBQX), and picrotoxin were obtained from Sigma-Aldrich. 6-cyano-7-nitroquinoxaline-2,3-dione (CNQX), AP-V, dl-TBOA, and d-serine were obtained from Tocris. NBQX, CNQX, and dl-TBOA were dissolved in dimethyl sulfoxide. Tetrodotoxin (TTX) was obtained from Abcam. NTG was obtained from Sigma-Aldrich. Picrotoxin was dissolved in ethanol (EtOH). AP-V, d-serine, and TTX were dissolved in ddH_2_O.

### Acute brain slice preparation

Mice were briefly anesthetized with isoflurane and decapitated. The brain was quickly removed and transferred to an ice-cold solution containing 65 mM NaCl, 2.5 mM KCl, 1.25 mM NaH_2_PO_4_, 25 mM NaHCO_3_, 7 mM MgCl_2_, 0.5 mM CaCl_2_, 25 mM glucose, and 105 mM sucrose saturated with 95% O_2_ and 5% CO_2_; coronal slices (350 μm thick) containing the ACC were cut from the tissue block with a vibratome (HM 650, Microm). Slices were then transferred to a recovery solution containing 130 mM K-gluconate, 15 mM KCl, 0.2 mM EGTA, 20 mM Hepes, and 25 mM glucose for 2 min before being kept in oxygenated ACSF (315 mosm) saturated with 95% O_2_ and 5% CO_2_ and containing 125 mM NaCl, 2.5 mM KCl, 1.25 mM NaH_2_PO_4_, 25 mM NaHCO_3_, 1 mM MgCl_2_, 2 mM CaCl_2_, and 25 mM glucose at 34°C for 25 min and then at room temperature until use. Slices used for two-photon glutamate imaging and astrocytic patch-clamp recordings were loaded with SR-101 (1 μM) for 15 min at 34°C before being kept in ACSF at room temperature.

### Electrophysiological recordings

#### Whole-cell recordings from astrocytes

Recordings were performed as previously described in (*17*). Individual slices were transferred to a recording chamber perfused with oxygenated ACSF, at a flow rate of 1 to 2 ml/min at 32° to 34°C. Whole-cell recordings were taken from L1 astrocytes in the Cg. Unless otherwise stated, cell bodies of astrocytes were visualized using an astrocyte-specific dye (SR-101; see above) that was excited with wLS broadband light-emitting diode illumination (460 nm), and images were acquired with Retiga R1 camera using Ocular software (QImaging, Germany) with a 40× water immersion objective. In addition, astrocytes were recognized by their hyperpolarized resting membrane potential, their linear current-voltage relationship, their inability to generate APs, and their low input resistance. Recordings were taken with borosilicate glass pipettes (4 to 8 megohm) containing the following internal solution: 115 mM K-gluconate, 6 mM KCl, 5 mM glucose, 7.8 mM Na-phosphocreatine, 4 mM Mg-ATP (adenosine triphosphate), 0.4 mM Na-GTP (guanosine triphosphate) [pH 7.25 with KOH; osmolarity, 295 mosm (readjusted with sucrose when necessary)]. Recordings were performed using MultiClamp 700B amplifier, and data were acquired with a Digidata 1550A 16-bit board (all from Molecular Devices). For the recording of STCs, the extracellular solution contained antagonists of NMDA receptors (AP-V; 50 μM), AMPA receptors (CNQX or NBQX; 10 μM), and γ-aminobutyric acid type A (GABA_A_) receptors (picrotoxin; 100 μM). Astrocytes were held at −80 mV, and STCs were evoked by single-pulse stimulation or by trains of 10 and 11 pulses at high frequencies (50 and then 100 Hz) every 20 s. The K^+^ inward current was characterized as the slowly decaying inward current elicited in astrocytes upon high-frequency synaptic stimulations. Every protocol was repeated at least five times and then averaged and analyzed. Currents were evoked by focal electrical stimulation (bipolar, 100 μs, 8.5 V) through a theta glass pipette placed in L1, in the proximity of the recorded astrocyte. Access resistance was monitored (<16 megohm), and recordings with an access resistance changing more than 30% between the beginning and the end of the recording were discarded. Resting membrane potential and input resistance were monitored for analysis of electrophysiological properties of astrocytes. The decay kinetics of the last pulse of the trains were analyzed by subtracting the current elicited by 10 pulses from that elicited by the 11th pulse.

#### Whole-cell recordings from L5 pyramidal neurons

Somas were patched with borosilicate glass pipettes (2.2 to 4 megohm). Cells were clamped at −70 mV, and focal synaptic stimulation was performed through a theta patch pipette located close to the selected apical tuft dendritic segments in L1. NMDA spikes were evoked by applying paired-pulse stimulations of 50 Hz of increasing stimulation intensities (from 1 to 2.5 mA or 5.5 mA). Recordings with an access resistance >15 megohm were discarded. The following internal solution was used: 130 mM K-gluconate, 5 mM KCl, 10 mM Hepes, 10 mM phosphocreatine, 4 mM Mg-ATP, 0.3 mM GTP, and biocytin (1.5 mg/ml) (pH 7.3 with KOH; osmolarity, 294 mosm). To visualize the dendrites, patch pipettes also contained Alexa Fluor 488. Recordings were performed in the presence of GABA_A_ receptor blocker (picrotoxin; 100 μM) and d-serine (10 μM).

For mEPSCs, somata were targeted for recording with borosilicate glass pipettes (2.2 to 4 megohm) containing the following: 130 mM gluconic acid, 130 mM CsOH, 5 mM CsCl, 10 mM Hepes, 1.1 mM EGTA, 10 mM Na-phosphocreatine, 4 mM Mg-ATP, and 0.3 mM Na-GTP. The pH of the intracellular solution was adjusted to 7.3 with CsOH, and biocytin (1.5 mg ml^−1^) was added for the reconstruction of neurons. Cells were held at −70 mV, and synaptic responses were recorded in the presence of picrotoxin (100 μM) and TTX (1 mM). Recordings with an unstable baseline or a holding current less than −400 pA were rejected. Currents were filtered off-line using a Butterworth low-pass filter (2 kHz) and analyzed in 1-min epochs using the Mini Analysis Program 6.0.7 (Synaptosoft Inc., USA). Recordings with leak current increasing more than 100 pA and access resistance changing more than 30% between the beginning and the end of the recording were discarded. At least 100 events were analyzed for every condition. Events were identified as mEPSCs by setting the event detection threshold at least twice the baseline noise level and by checking that events had (i) rise times faster than the decay time, (ii) rise times greater than 0.5 ms, and (iii) decay times greater than 1.5 ms. Events not fitting the above parameters were rejected. Event amplitudes, interevent intervals, and rise and decay times were first averaged within each experiment and regrouped by condition. The resulting means were averaged between experiments. Single-cell properties (access resistance, membrane capacitance, etc.) were analyzed with Clampfit 10.5 (Axon Instruments, Union City, CA). Graphs were made using GraphPad Prism and Illustrator 15.1.0 (Adobe). For evoked AMPA-mediated excitatory postsynaptic currents (eEPSCs), cells were held at −70 mV and focal synaptic stimulation was performed through a theta patch pipette located close to the tuft dendrites in L1. AMPA eEPSCs were evoked by applying either a single stimulation or paired-pulse stimulations at 20 Hz. Recordings were acquired in the presence of picrotoxin (100 μM) and AP-V (50 μM) in ACSF.

### Biocytin labeling

For some experiments, internal solutions used for recording of pyramidal cells contained biocytin (1.5 mg/ml) that diffused in the cells for at least 10 min, as previously described in (*17*). Briefly, slices (350 μm) containing the recorded cell were then fixed in 4% paraformaldehyde (PFA) at +4°C overnight. The following day, slices were washed in phosphate-buffered saline (PBS) and 274 mM NaCl before being transferred to a blocking solution containing 10% normal goat serum in 0.3% Triton-PBS and 274 mM NaCl for 1 hour. Afterward, slices were put in a blocking solution containing Alexa Fluor 647–conjugated streptavidin (1:700; Jackson ImmunoResearch Europe Ltd.; code: 016-600-084) for 2 hours. Slices were then washed in PBS and 274 mM NaCl before being mounted on Superfrost Plus slides. Images were acquired on a Zeiss LSM 710 Pascal confocal microscope using a 0.9 numerical aperture ×10 Plan-Apochromat objective (for L5 pyramidal cells) and the ZEN 2012 software (Carl Zeiss). Whenever applicable, contrast and illumination were adjusted in ImageJ. Presented images are *Z* projections.

### AAV5/2 generation and injections in vivo

The *mAtp1a2* gene (WT form of α2 NKA for compensation experiments) and the *EGFP* gene (for control experiments) were cloned into plasmid backbones containing a shorted glial fibrillary acidic protein (HgfaABC1D) promoter as in (*22*). These plasmids were packaged into AAV serotype 5 [AAV-5/2-hGFAP-mATP1A2-bGHp(A) and AAV5/2- hGFAP-hHBb1/E-EGFP-bGHp(A)] by the Viral Vector Facility of the University of Zurich. All electronic information concerning the plasmids and viruses is available online in the viral vector repository (https://vvf.ethz.ch). FHM2 KI mice were used in all experiments in accordance with institutional guidelines.

All surgical procedures were conducted under general anesthesia using continuous isoflurane inhalation (induction at 5% and maintenance at 1 to 3%). Following induction of anesthesia, the mice were placed into a motorized stereotaxic frame (David Kopf Instruments and NeuroStar) using adjustable ear bars and an anesthesia mask with an incisor bar. The animal was maintained at a physiological temperature using a heating mat placed between the animal and the frame. Vitamin C ointment was applied to the eyes to prevent corneal drying during the operation. Mice were subcutaneously administered buprenorphine (0.2 mg/kg) before surgery. The fur between the ears was shaved, scrubbed with EtOH and betadine, and dried, and a longitudinal incision of approximately 3 to 5 mm was made in the skin above the skull. For unilateral injections, a single hole was drilled through the skull directly above the Cg on one hemisphere (stereotaxic coordinates with respect to bregma: 0.75 mm anterior, 0.3 mm lateral, and 1.25 mm ventral). For control experiments, 0.5 μl of the control virus AAV5/2- hGFAP-hHBb1/E-EGFP-bGHp(A) was injected through a glass pipette. Since AAV vectors size is limited to ~4.7 kb, it was not possible to include a reporter gene in the rescue virus AAV-5/2-hGFAP-mATP1A2-bGHp(A). Therefore, to visualize the injection site, we injected a mixture of 0.5 μl of the two viruses with a ratio of 2:1 of the rescue virus to the control virus containing eGFP. Glass pipettes were left in place for at least 10 min following infusion of the virus. Surgical wounds were closed with single 5-0 nylon sutures. Following surgery, animals were closely monitored. Mice were euthanized 15 to 25 days after surgery for electrophysiology experiments or were used for behavioral experiments. When assessing behavior, AAVs were injected bilaterally. The experimenter was blinded to which AAV was injected.

### Two-photon glutamate imaging

Mice aged between 4 and 6 weeks were injected with 0.3 to 0.5 μl of AAV2/1.GFAP.iGluSnFr.WPRE.SV40 (Penn Vector; provided by L. Looger, Janelia Farm) unilaterally into the Cg through a glass pipette as described above. Fifteen to 21 days following the virus injections, coronal brain slices (350 μm) containing the Cg were obtained as previously described (*21*). Imaging was performed as described in (*17*). Briefly, a galvanometer-based two-photon laser scanning system was used to image extracellular glutamate (16× objective; zoom, 6; excitation wavelength, 900 nm; 64pixels by 64 pixels per image; acquisition rate, 19.2 Hz). Astrocytes were visualized using SR-101. Synaptic glutamate release was elicited by trains of 10 pulses at high frequency (50 or 100 Hz) every 20 s delivered via a theta glass pipette (bipolar, 100 μs; stimulation intensity, 3 to 5 V) placed in the inner L1. To visualize the theta glass pipette, it was filled with ACSF containing 1 μM SR-101. Note that SR-101 staining does not affect the kinetics of iGluSnFr transients nor of STCs as shown in (*17*). Moreover, when we doubled the acquisition rate to 38.4 Hz, the iGluSnFr decay kinetics remained unaltered, indicating that the image acquisition rate used was sufficient to detect the monitored changes. All solutions contained 10 μM NBQX or CNQX, 50 μM AP-V, and 100 μM picrotoxin, and temperature was kept between 32° and 34°C during imaging. Ten consecutive sweeps were acquired and subsequently analyzed using ImageJ. Fluorescence emission was collected from an ROI (diameter, 34 μm) 10 to 40 μm away from the stimulation pipette. The average background value was derived from a region within the field of view that was free of clearly visible iGluSnFr (typically in the contralateral hemisphere) and subtracted from the fluorescence intensity of the ROIs for each frame. Traces were then averaged, and decay tau was calculated by fitting a single exponential function using Igor Pro (WaveMetrics).

### Behavioral analysis

#### Orofacial von Frey

Behavioral testing was performed in a dimly lit and quiet room by the same female experimenter. Throughout the week before the first behavioral testing, mice were habituated to handling by the experimenter. Each mouse was placed in Plexiglas cages of about 20 cm by 20 cm sitting on a metal grid ground floor. Mice were allowed to accommodate to the cage for 1 hour before the testing. In all experiments, 0.025- and 0.01-g von Frey filaments were used. The first tests were performed using the lower-force filament. Filaments were applied to the orofacial area close to the whisker pad or close to a 90° angle until bent, in three series of four pokes, in the middle or on either left or right side of the snout. After a total of 12 pokes with the 0.025-g filament, the stimulation with the 0.01-g filament was applied in the same way. The responses recorded were as follows: unilateral or bilateral forepaw swipes across the face (1 point each), continuous forepaw swipes (three or more: 1.5 points), aggression/biting of the probe following stimulus (0.25 points), and clear withdrawal of the head from the stimulus (0.25 points) as described in (*27*). Before the injection of NTG (5 or 10 mg/kg) or 0.9% saline (intraperitoneally), baseline 1 was recorded several hours or a day before the experiment and baseline 2 was immediately before intraperitoneal injections. Testing was carried out at 30, 60, 120, and 180 min after injection. The experimenter was blinded to the treatment in all behavioral experiments.

#### Locomotor activity

NTG (5 mg/kg, i.p.) or saline was administered 30 min before testing. Locomotor activity was measured in an open-field arena (radius, 10 cm) equipped with four pairs of light beams and photosensors and analyzed for the time interval between 10 and 60 min after NTG or saline administration.

### Brain clearing and light-sheet microscopy

#### After fixation and dehydration

Mice were anesthetized with isoflurane and decapitated. Entire brains were extracted, briefly washed in PBS to remove excessive blood, and postfixed in 4% PFA for 2 days at 4°C. After fixation, brains were washed two times in PBS and subsequently dehydrated in increasing alcohol concentrations [30, 50, 70, 80, 90, 96, and 100% EtOH (each adjusted to pH 9.5)] for a day each (*39*). Tissue shrinkage of up to 50% was observed during the dehydration process. After dehydration, brains were transferred into a clearing solution of benzyl alcohol and benzyl benzoate (1:2) solution (BABB) in separate glass vials on a gentle shaking or rotating cycle under a chemical hood for a minimum of 1 day until transparent.

#### Imaging

Cleared brains were transferred to an immersion cuvette containing BABB and placed in the imaging reservoir of the microscope. Images were acquired using the mesoscale single-plane illumination microscope mesoSPIM system (www.mesospim.org) with an Olympus MVX10 macroscope in the detection path and an MVPLAPO 1× objective (*40*). The fluorescent signal in the sample was recorded at ×1.6 magnification by moving the sample through the light sheet in 4-μm steps. The sample was illuminated from one side using Toptica multi-laser engine laser at excitation wavelengths of 488 and 640 nm. Postprocessing of images was carried out using ImageJ and Imaris software (Bitplane).

### Western blots

Two weeks following stereotaxic viral injections in the Cg, tissue of the Cg was rapidly dissected and immediately frozen on dry ice and stored at −80°C until used. On the day of the experiment, the tissue (10 to 20 mg) was thawed and homogenized by sonication in 20 volumes of 20 mM tris (pH 7.4) containing the protease inhibitor cocktail cOmplete Mini (Roche Diagnostics). The homogenate was centrifuged for 10 min at 1000*g*, and the supernatant was recovered. After total protein quantification using the Bradford protein assay (Bio-Rad), the samples were incubated with Laemmli sample buffer (Bio-Rad) for 30 min at 37°C. Aliquots containing 10 μg (GLAST) or 15 μg of protein (ATPase α2 and GLT-1) were subjected to SDS–polyacrylamide gel electrophoresis using 10% (GLAST and GLT-1) or 7.5% (ATPase α2) mini gels (Mini-PROTEAN 3, Bio-Rad). Proteins were transferred onto nitrocellulose membranes in a Trans-Blot semi-dry transfer cell (Bio-Rad) at 15 V for 60 min using 39 mM glycine, 48 mM tris, 1.3 mM SDS, and 20% methanol as transfer buffer. After blotting, the transferred proteins were stained with REVERT Total Protein Stain (LI-COR) and immediately imaged using the Odyssey CLx imager (LI-COR). For immunodetection, the blots were blocked for 1 hour in PBS containing 5% nonfat dry milk at room temperature, followed by incubation at 4°C overnight with anti–α_2_ NKA antibodies (1:1000; rabbit polyclonal, Sigma-Aldrich, catalog no. 07-674), anti–GLT-1 antibodies (1:1000; rabbit polyclonal, knockout verified, Synaptic Systems, catalog no. 250203), or anti-GLAST antibodies (1:10,000; rabbit polyclonal, knockout verified, Synaptic Systems, catalog no. 250113), diluted in PBST [PBS (pH 7.4) and 0.05% Tween 20] containing 5% nonfat dry milk. The blots were then washed five times for 5 min with Tris Buffered Saline + Tween 20 (TBST) (10 mM Tris, pH 7.4, 150 mM NaCl, 0.05% Tween 20) and incubated with secondary antibodies (1:20,000; goat anti-rabbit Alexa Fluor Plus 800) for 1 hour at room temperature. Following extensive washing (see above), immunoreactivity was detected using the Odyssey CLx imager (LI-COR). Immunoreactivity was quantified with the Image Studio software (LI-COR) and normalized to total protein in the corresponding lanes. Each antibody was tested for its linear detection range using different protein and antibody concentrations.

### Immunohistochemistry and image analysis

Three weeks after stereotaxic viral injections in the Cg, FHM2 KI mice were anaesthetized with pentobarbital (160 mg kg^−1^, i.p.) before transcardiac perfusion with 20 ml of phosphate buffer followed by 100 ml of 4% ice-cold PFA [in 0.1 M sodium phosphate buffer (pH 7.4)]. Brain tissue was postfixed for 4 hours with 4% PFA on ice and cryoprotected in 30% sucrose solution (in 0.1 M sodium phosphate buffer) overnight at 4°C. Brains were embedded in NEG50 frozen section medium (Richard-Allan Scientific) and cut into 40-μm free-floating sections (Hyrax KS 34 microtome, Carl Zeiss). Brain sections were left in antifreezing solution at −20°C until use. Following incubation in blocking solution (PBS, 0.3% Triton X-100, and 10% normal donkey serum) for 1 hour, brain sections were incubated at 4°C overnight in a primary antibody solution (PBS, 0.3% Triton X-100, and 10% normal donkey serum) containing combinations of the following antibodies: chicken anti-GFP (1:1000; LifeTech, AB_2534023), guinea pig anti-NeuN (1:1000; Synaptic Systems, AB_2619988), and rabbit anti-S100B (1:700; Abcam, AB_52642). Three washing steps of 10 min each in PBS were performed before incubating brain sections with secondary antibodies (1:500): Cyanine Cy3 donkey anti-rabbit (Jackson ImmunoResearch, AB_2307443), Alexa Fluor 647 donkey anti-guinea pig (Jackson ImmunoResearch, AB_2340477), and Alexa Fluor 488 donkey anti-chicken (Jackson ImmunoResearch, AB_2340376) for 90 min at room temperature in PBS supplemented with 0.3% Triton X-100. Images were taken with an LSM 800 with Airyscan confocal microscopes (Carl Zeiss) controlled with ZEN 2.3 (blue edition) software and using a Plan-Apochromat ×40/1.4 Oil DIC M27 oil-immersion objective. *Z* stack images of 10 optical sections and 1.5-μm step size were used for the analysis of fluorescence colocalization and to create maximum intensity projections images. Images were processed using ImageJ software.

### Quantification and statistical analysis

Data are displayed as means ± SEM in all experiments except for the Western blot data that are displayed as means ± SD. Statistical details can be found in Results, figures, and figure legends. Statistical comparisons were made with two-tailed paired or unpaired *t* tests, one-way analysis of variance (ANOVA) with Bonferroni post hoc test, one-way ANOVA with Tukey post hoc test, or two-way repeated-measures ANOVA test. All graphs and statistical tests were performed using GraphPad Prism, and figures were prepared using Adobe Illustrator CS5. *P* values less than 0.05 were considered statistically significant.

## Supplementary Material

aaz1584_SM.pdf

## SUPPLEMENTARY MATERIALS

Supplementary material for this article is available at http://advances.sciencemag.org/cgi/content/full/6/23/eaaz1584/DC1

## Appendix

View/request a protocol for this paper from *Bio-protocol*.
