# Supplementary material for: Astrocyte dysfunction increases cortical dendritic excitability and promotes cranial pain in familial migraine
Source: Sci Adv. 2020 Jun 5;6(23):eaaz1584. doi: 10.1126/sciadv.aaz1584 (PMC7274778; doi:10.1126/sciadv.aaz1584)
Supplement: aaz1584_SM.pdf [file aaz1584_SM.pdf]

Science Advances
AAAS

advances.sciencemag.org/cgi/content/full/6/23/eaaz1584/DC1

Supplementary Materials for

Astrocyte dysfunction increases cortical dendritic excitability and promotes cranial pain in familial migraine

Jennifer Romanos, Dietmar Benke, Daniela Pietrobon, Hanns Ulrich Zeilhofer, Mirko Santello*

*Corresponding author. Email: mirko.santello@pharma.uzh.ch

Published 5 June 2020, *Sci. Adv.* **6**, eaaz1584 (2020)
DOI: 10.1126/sciadv.aaz1584

This PDF file includes:

Figs. S1 to S10

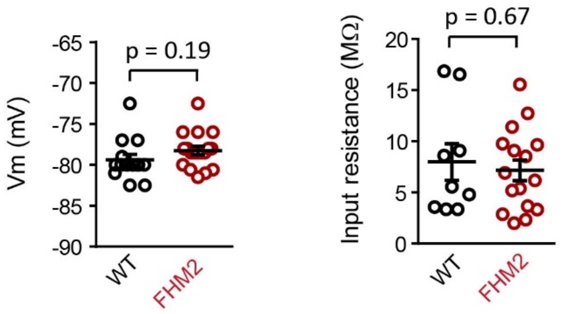

Fig. S1. Astrocytic resting membrane potential and input resistance in WT and FHM2 mice. *Left* Comparison of resting membrane potential in Cg astrocytes of WT and FHM2 KI mice (WT: Vm = -79.39 ± 0.7 mV n =14 cells, FHM2 KI: Vm = -78.26 ± 0.5 mV, n=18 cells, p = 0.19). *Right* Comparison of input resistance in Cg astrocytes of WT and FHM2 KI mice (WT: Rn= 7.96 ± 1.8 MOhm, *n* = 9 cells, FHM2 KI: Rn = 7.16 ± 1.0 MOhm, *n* = 16 cells, *P* = 0.67). Data are mean ± SEM. *n* = number of cells. Two-tailed unpaired *t* test.

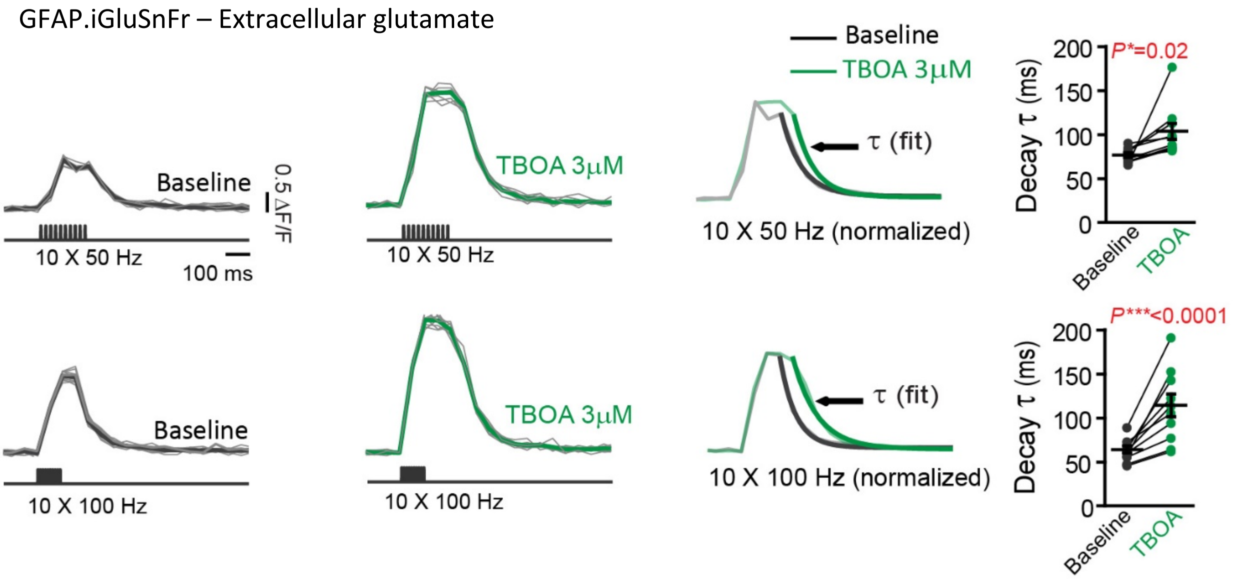

Fig. S2. Partial blockade of glutamate transporters enhances extracellular glutamate. Upon trains of synaptic stimulation: 10x50 Hz and 10X100 Hz, robust and consistent increases in iGluSnFr emission could be detected. Thick lines represent the average of the responses and the mono-exponential fit of the decay. The decay kinetics of the averaged transients are significantly affected by partial blockade of GluTs by application of 3 μM TBOA following 50 Hz and 100 Hz stimulation. (50 Hz: Baseline τ_{decay} = 75.62 ± 2.55 ms, P = 0.44; TBOA τ_{decay} = 102.7 ± 9.09 ms, n = 10, P* = 0.02. 100 Hz: Baseline τ_{decay} = 62.13 ± 3.99 ms; TBOA τ_{decay} = 112.31 ± 13 ms, n = 10, P** = 0.0008). Representative traces are the average of at least 5 sweeps. Data are mean ± SEM. n = number of slices. Two-tailed paired t test.

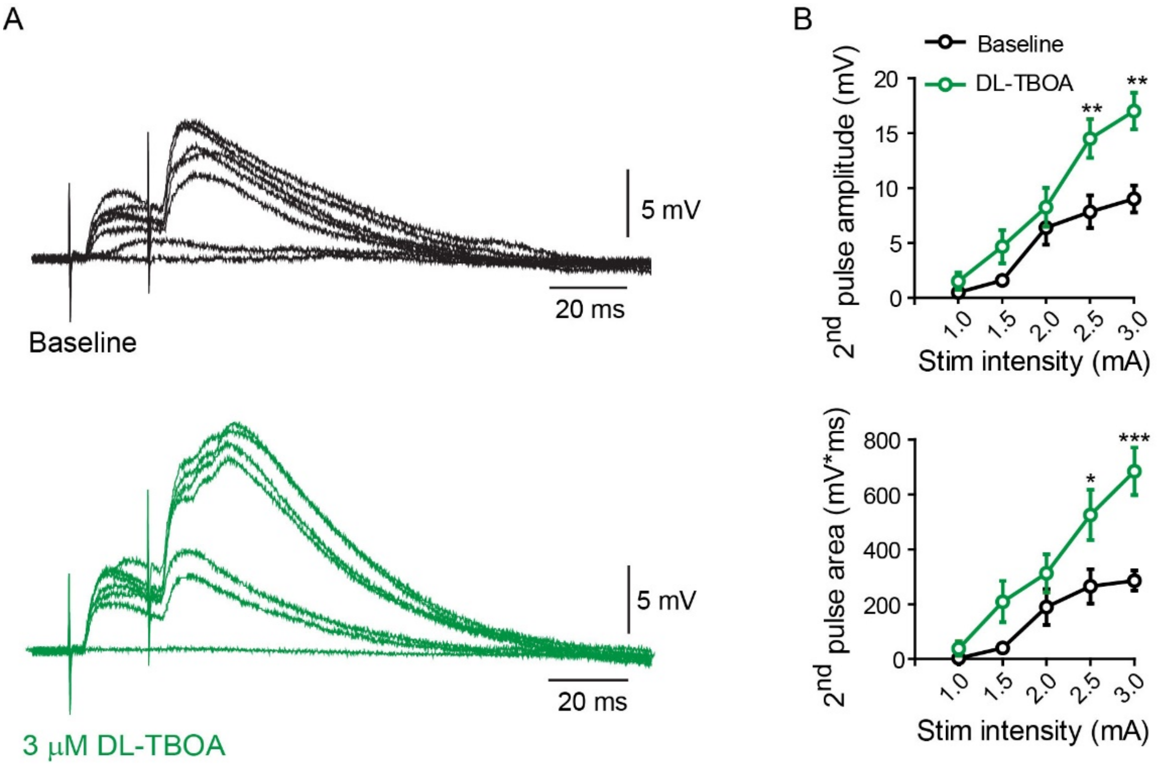

Fig. S3. Partial blockade of glutamate transporters directly enhances NMDA spikes generation. **(A)** Representative traces of NMDA spikes evoked by focal synaptic stimulation (paired-pulse, 50 Hz) of increasing stimulation intensities that cause an abrupt and non-linear increase in amplitude and area under the curve (AUC) of the second pulse, which is characteristic of NMDA spikes in baseline conditions (black) and in the presence of 3 µM DL-TBOA (green). **(B)** The amplitude and the AUC of the 2nd pulse are significantly higher in the presence of subsaturating concentrations of DL-TBOA (3 µM) compared to baseline (2nd pulse amplitude: Baseline 7.8 ± 1.7 mV; DL-TBOA 14.5 ± 2.94 mV, n = 7 cells, N = 3 mice; $P^{**} < 0.01$. 2nd pulse AUC: Baseline 264.5 ± 58 mV·ms; DL-TBOA 525.2 ± 114 mV·ms, n = 7; $P^{*} < 0.05$; Stimulation intensity 2.5 mA). Data are mean ± SEM. 2-way ANOVA with Bonferroni post-hoc test.

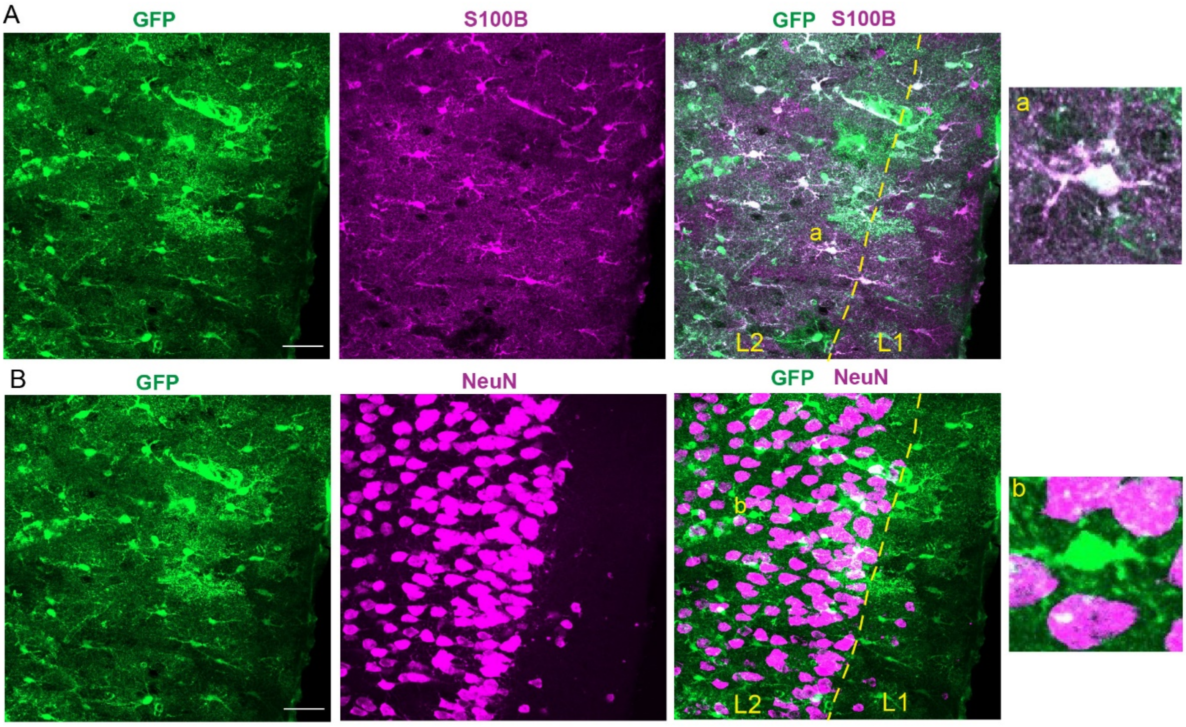

Fig. S4. The viral vectors used in the rescue experiments specifically target astrocytes. (A) Coronal section of the cingulate cortex of FHM2 mouse injected with a mixture of rescue and control virus containing GFP and immunostained against an astrocytic marker S100B (magenta) and against GFP, the reporter used in the viral vector (green). These experiments show that GFP and S100B extensively overlap with each other **(B)** Same as **(A)** but immunostained against a neuronal marker NeuN (magenta) and against GFP. There was no overlap of NeuN and GFP observed, pointing towards non-neuronal infection. Objective 40X, scale bars = 40 µm.

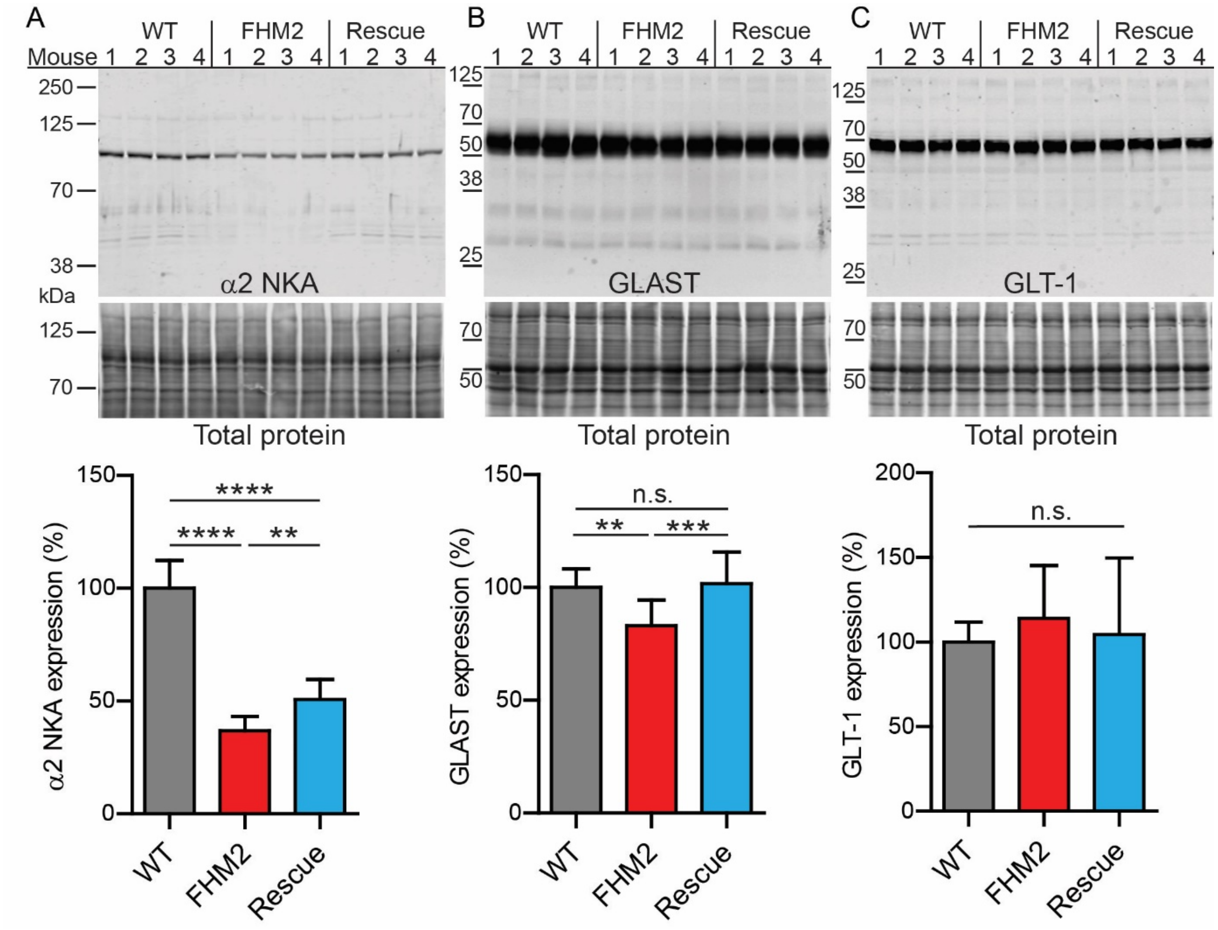

Fig. S5. α2 NKA, GLAST and GLT-1 expression in the cingulate cortex of WT, FHM2 and FHM2 rescue mice. Representative western blots derived from 4 individual mice are shown in the top panels for **(A)** α2 NKA, **(B)** GLAST and **(C)** GLT-1. The bottom panels: corresponding staining for total blotted protein used for normalization shown. For quantification, band intensities were normalized to total protein in the corresponding lanes. The bar graphs depict the relative expression of α2 NKA, GLAST and GLT-1 in WT (set to 100%), FHM2 and FHM2 mice injected with AAV.ATP1A2 (Rescue) virus. Data are the mean ± SD derived from 4 individual mice and 3 repetitions. 1-way ANOVA with Tukey post hoc test; n.s. $P > 0.05$, P**< 0.025, P***< 0.0008, P****< 0.0001.

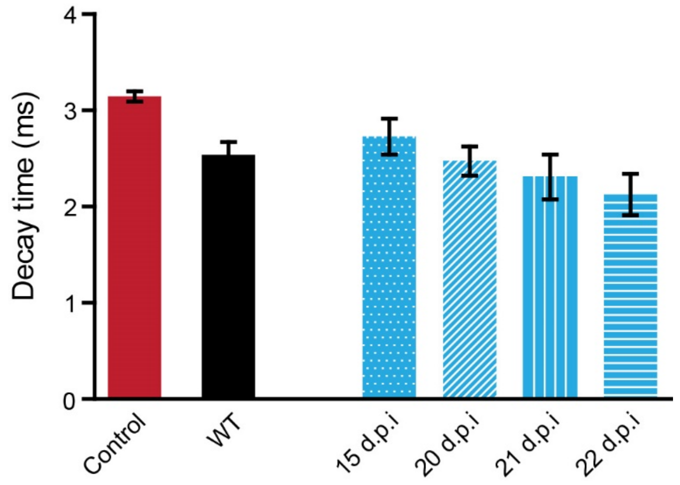

Fig. S6. Values of STC decay tau in FHM2 mice injected with rescue virus at different days post injection. STC decay kinetics at 100 Hz in FHM2 mice injected with the rescue virus (in blue) at 15, 20, 21 and 22 days post injection compared to STC decay times of WT mice (black) and FHM2 KI mice injected with the control virus (red).

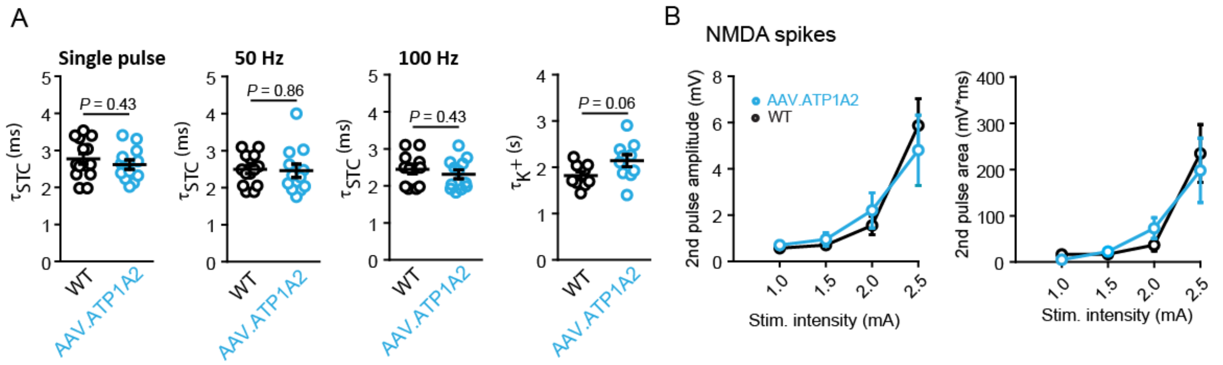

Fig. S7. Decay kinetics of STCs in astrocytes and NMDA spikes in rescued FHM2 mice compared to WT mice. (A) The average STCs decay time elicited by single pulse, and by the 11th pulse of 50 Hz and of 100 Hz trains in FHM2 mice injected with the hGFAP.ATP1A2 (rescue virus) became similar to STC decay kinetics in WT mice. Each point represents the STC decay time in one astrocyte. (Single pulse: WT $\tau_{decay} = 2.77 \pm 0.14$ ms, *n* = 14 cells, FHM2 Rescue $\tau_{decay} = 2.62 \pm 0.13$ ms, *n* = 12, *P* = 0.43. 50 Hz: WT $\tau_{decay} = 2.49 \pm 0.11$ ms; FHM2 Rescue $\tau_{decay} = 2.46 \pm 0.18$ ms, *P* = 0.86. 100 Hz: WT $\tau_{decay} =$ 2.45 ± 0.12 ms, FHM2 rescue $\tau_{decay} = 2.31 \pm 0.12$ ms, *n* = 22, *P* = 0.43). Similarly, the decay kinetics of K^+ currents in rescued FHM2 mice became similar to WT levels (WT $\tau_{decay} = 1.82 \pm 0.08$ s, *n* = 9; FHM2 rescue $\tau_{decay} = 2.14 \pm 0.13$ s, *n* = 10, *P* = 0.06). **(B)** The amplitude and the AUC of the 2nd pulse in rescued FHM2 mice became comparable to the values observed in WT mice. Data are mean ± SEM. *n* = number of cells. Two-tailed unpaired *t* test.

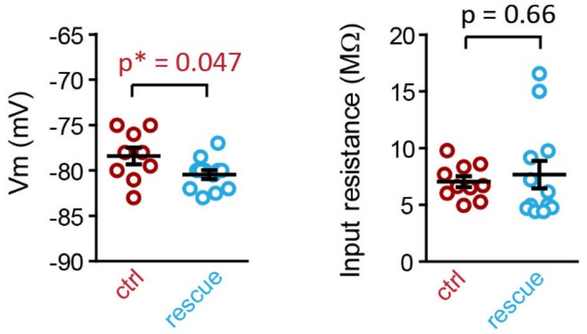

Fig. S8. Astrocytic resting membrane potential and input resistance FHM2 mice injected with rescue and control virus. *Left* Comparison of resting membrane potential in Cg astrocytes of FHM2 KI mice injected with control virus (red) or rescue virus (blue) (Control: Vm = -78.39 ± 0.9 mV *n* = 9 cells, rescue: Vm = -80.46 ± 0.5 mV, *n* = 12 cells, *P** = 0.04). *Right* Comparison of input resistance in Cg astrocytes of WT and FHM2 KI mice (Control: R_n= 7.05 ± 0.5 MOhm, *n* = 10 cells, Rescue: R_n = 7.68 ± 1.2 MOhm, *n* = 12 cells, *P* = 0.66). Data are mean ± SEM. *n* = number of cells. Two-tailed unpaired *t* test.

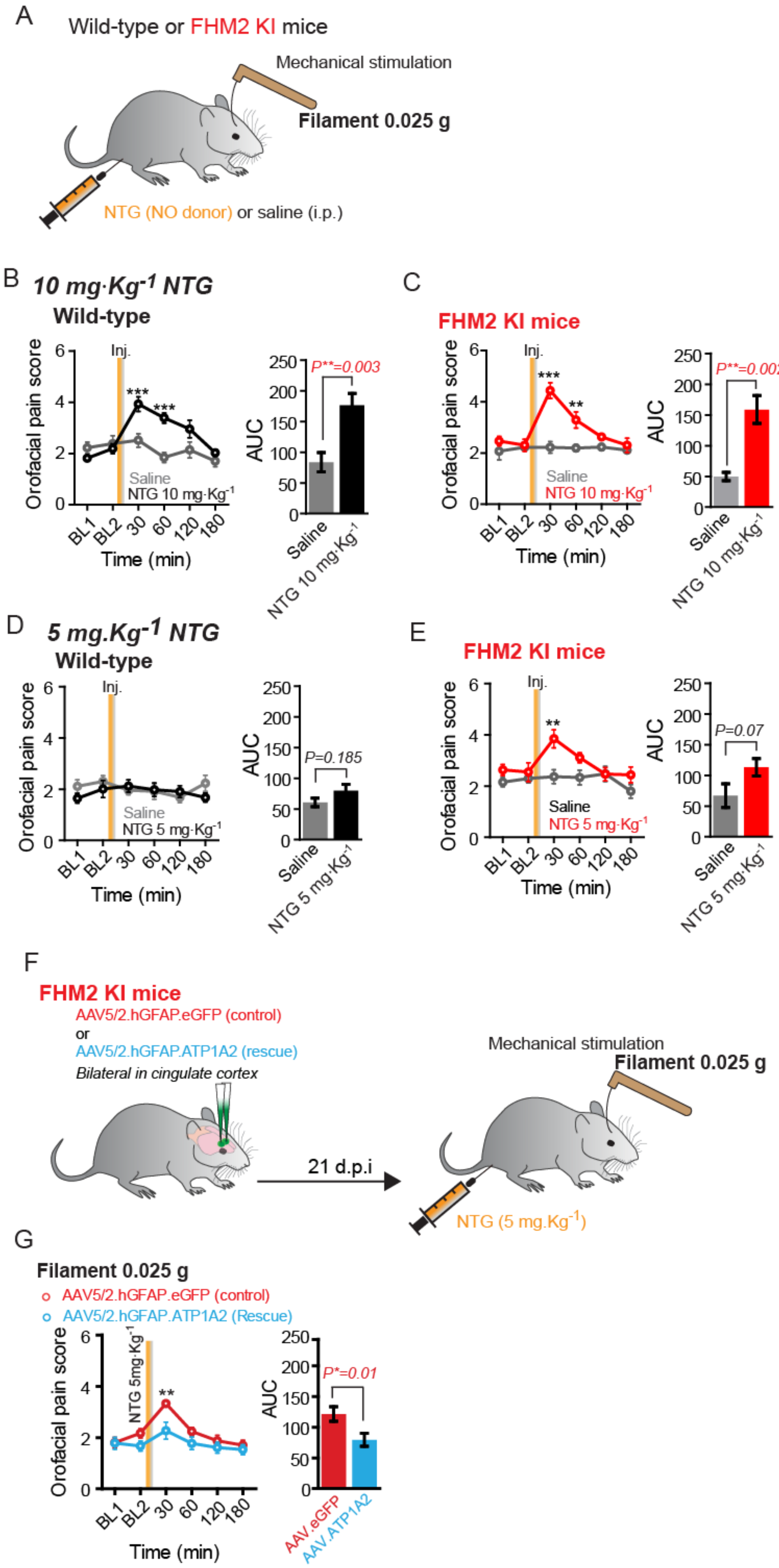
A
Wild-type or FHM2 KI mice
Mechanical stimulation
Filament 0.025 g
NTG (NO donor) or saline (i.p.)
B
10 mg·Kg-1 NTG
Wild-type
Orofacial pain score
Inj.
***
***
Saline
NTG 10 mg·Kg-1
BL1 BL2 30 60 120 180
Time (min)
AUC
P**=0.003
Saline
NTG 10 mg·Kg-1
C
FHM2 KI mice
Orofacial pain score
Inj.
***
**
Saline
NTG 10 mg·Kg-1
BL1 BL2 30 60 120 180
Time (min)
AUC
P**=0.002
Saline
NTG 10 mg·Kg-1
D
5 mg.Kg-1 NTG
Wild-type
Orofacial pain score
Inj.
Saline
NTG 5 mg·Kg-1
BL1 BL2 30 60 120 180
Time (min)
AUC
P=0.185
Saline
NTG 5 mg·Kg-1
E
FHM2 KI mice
Orofacial pain score
Inj.
**
Saline
NTG 5 mg·Kg-1
BL1 BL2 30 60 120 180
Time (min)
AUC
P=0.07
Saline
NTG 5 mg·Kg-1
F
FHM2 KI mice
AAV5/2.hGFAP.eGFP (control)
or
AAV5/2.hGFAP.ATP1A2 (rescue)
Bilateral in cingulate cortex
21 d.p.i
Mechanical stimulation
Filament 0.025 g
NTG (5 mg.Kg-1)
G
Filament 0.025 g
AAV5/2.hGFAP.eGFP (control)
AAV5/2.hGFAP.ATP1A2 (Rescue)
Orofacial pain score
NTG 5mg·Kg-1
**
BL1 BL2 30 60 120 180
Time (min)
AUC
P*=0.01
AAV.eGFP
AAV.ATP1A2

Fig. S9. Local astrocyte dysfunction in the Cg influences orofacial pain in FHM2 mice. (A) Similar to Fig 5 using a lighter von frey filament of 0.025 g. Schematic illustration of the experimental design to test orofacial mechanical hypersensitivity after injection of nitroglycerine or saline solution (i.p.) in wild-type (WT) or FHM2 mice. **(B)** Time-course showing the orofacial pain score in WT mice injected with either saline (grey) or NTG (black). 10 $mg \cdot Kg^{-1}$ NTG elicits hypersensitivity to touch at 30 and 60 mins following injection, which is reflected by a significantly higher area under the curve of the response. (AUC: WT: saline 83.80 ± 15.6 $N = 7$ mice, NTG 176.4 ± 19.25 $N = 6$, $P^{**} = 0.003$). **(C)** Same as **(B)** but for FHM2 mice injected with saline (grey) or NTG (red). 10 $mg.Kg^{-1}$ NTG evokes higher orofacial pain scores at 30 and 60 mins following injection, which is reflected by a significantly higher area under the curve of the response (FHM2: saline 49.91 ± 6.77 $N = 6$, NTG 158.8 ± 22.6 $N = 8$, $P^{**} = 0.0017$). **(D)** A lower dose of NTG 5 $mg.Kg^{-1}$ does not elicit a higher sensitivity in WT mice. **(E)** In FHM2 mice, 5 $mg \cdot Kg^{-1}$ NTG evokes a higher orofacial pain score at 30 mins post-injection. (AUC: WT: saline 60.59 ± 7.12 $N = 6$ mice, NTG 79.55 ± 10.33 $N = 8$, $P = 0.18$. FHM2: saline 67.02 ± 19.16 $N = 7$, NTG 113.2 ± 14.6 $N = 7$, $P = 0.07$). **(F)** Schematic illustration of the experimental design to test orofacial mechanical hypersensitivity induced by NTG (5 $mg \cdot Kg^{-1}$), 21 days after bilateral injection of AAV5/2.hGFAP.eGFP (control virus) or AAV5/2.hGFAP.ATP1A2 (rescue virus) in the cingulate cortex of FHM2 mice using a lighter von Frey filament (0.025 g). **(G)** Time-course showing the orofacial pain score in FHM2 mice injected with the control virus (red) or the rescue virus (blue). 5 $mg \cdot Kg^{-1}$ NTG only evokes a higher orofacial pain score at 30 mins post-injection in control FHM2 mice and not in rescued FHM2. This is reflected by a significantly lower area under the curve of the response in rescued mice. (AUC: control virus: 121.6 ± 11.92 $N = 14$ mice, rescue virus: 79.38 ± 10.53 $N = 13$, $P^{*} = 0.01$). Data are mean ± SEM. 2-way ANOVA with Bonferroni post-hoc test and Two-tailed unpaired *t* test.

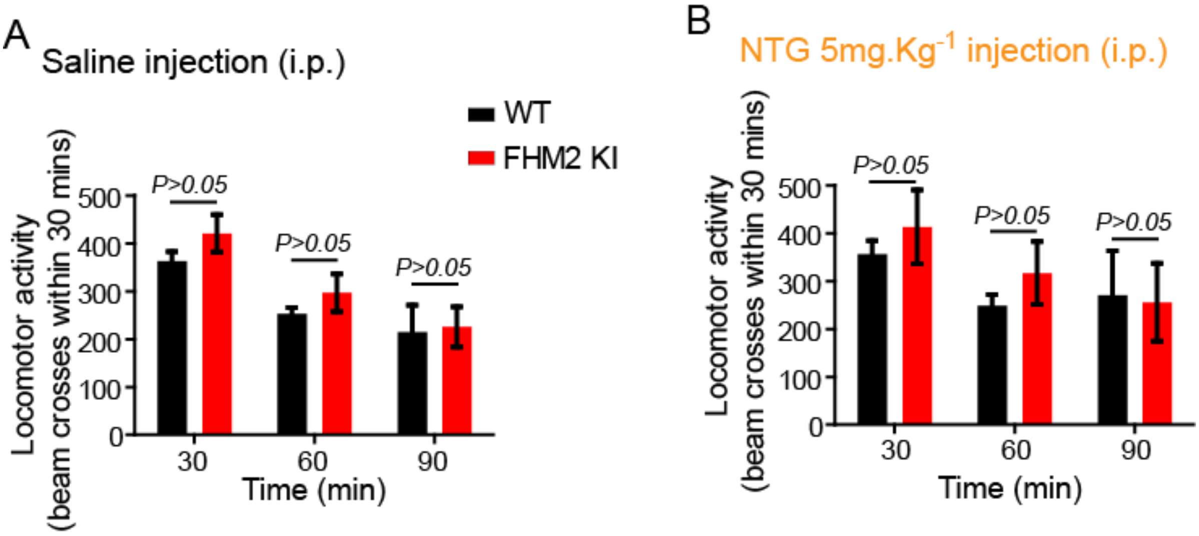

Fig. S10. Locomotor activity is not altered between FHM2 and WT mice upon NTG injection. Bar graphs presenting the mean locomotor activity in WT mice and FHM2 mice 30, 60 and 90 mins following the injection of either saline (left) or 5 $mg.Kg^{-1}$ NTG (right). Data are mean ± SEM. 2-way ANOVA with Bonferroni post-hoc test.
